# POP1 inhibits MSU-induced inflammasome activation and ameliorates gout

**DOI:** 10.3389/fimmu.2022.912069

**Published:** 2022-09-26

**Authors:** Lucia de Almeida, Savita Devi, Mohanalaxmi Indramohan, Qi-Quan Huang, Rojo A. Ratsimandresy, Richard M. Pope, Andrea Dorfleutner, Christian Stehlik

**Affiliations:** ^1^ Division of Rheumatology, Department of Medicine, Feinberg School of Medicine, Northwestern University, Chicago, IL, United States; ^2^ Department of Academic Pathology, Cedars Sinai Medical Center, Los Angeles, CA, United States; ^3^ Department of Biomedical Sciences, Cedars Sinai Medical Center, Los Angeles, CA, United States; ^4^ The Kao Autoimmunity Institute, Cedars Sinai Medical Center, Los Angeles, CA, United States; ^5^ Samuel Oschin Comprehensive Cancer Institute, Cedars Sinai Medical Center, Los Angeles, CA, United States

**Keywords:** inflammasome, gout, caspase-1, inflammation, macrophage, pyrin domain

## Abstract

Canonical inflammasomes are innate immune protein scaffolds that enable the activation of inflammatory caspase-1, and subsequently the processing and release of interleukin (IL)-1β, IL-18, and danger signals, as well as the induction of pyroptotic cell death. Inflammasome assembly and activation occurs in response to sensing of infectious, sterile and self-derived molecular patterns by cytosolic pattern recognition receptors, including the Nod-like receptor NLRP3. While these responses are essential for host defense, excessive and uncontrolled NLRP3 inflammasome responses cause and contribute to a wide spectrum of inflammatory diseases, including gout. A key step in NLRP3 inflammasome assembly is the sequentially nucleated polymerization of Pyrin domain (PYD)- and caspase recruitment domain (CARD)-containing inflammasome components. NLRP3 triggers polymerization of the adaptor protein ASC through PYD-PYD interactions, but ASC polymerization then proceeds in a self-perpetuating manner and represents a point of no return, which culminates in the activation of caspase-1 by induced proximity. In humans, small PYD-only proteins (POPs) lacking an effector domain regulate this key process through competitive binding, but limited information exists on their physiological role during health and disease. Here we demonstrate that POP1 expression in macrophages is sufficient to dampen MSU crystal-mediated inflammatory responses in animal models of gout. Whether MSU crystals are administered into a subcutaneous airpouch or into the ankle joint, the presence of POP1 significantly reduces neutrophil infiltration. Also, airpouch exudates have much reduced IL-1β and ASC, which are typical pro-inflammatory indicators that can also be detected in synovial fluids of gout patients. Exogenous expression of POP1 in mouse and human macrophages also blocks MSU crystal-induced NLRP3 inflammasome assembly, resulting in reduced IL-1β and IL-18 secretion. Conversely, reduced POP1 expression in human macrophages enhances IL-1β secretion. We further determined that the mechanism for the POP1-mediated inhibition of NLRP3 inflammasome activation is through its interference with the crucial NLRP3 and ASC interaction within the inflammasome complex. Strikingly, administration of an engineered cell permeable version of POP1 was able to ameliorate MSU crystal-mediated inflammation *in vivo*, as measured by neutrophil infiltration. Overall, we demonstrate that POP1 may play a crucial role in regulating inflammatory responses in gout.

## Introduction

Gout is a common form of inflammatory arthritis which usually affects a single joint and is characterized by recurrent inflammatory flares. It is caused by hyperuricemia that triggers monosodium urate (MSU) crystal formation, and MSU deposition into joints triggers a local inflammatory response. Recent evidence also exists for a contribution of gut dysbiosis and a resulting intestinal integrity impairment to the pathogenesis of gout in humans and mice (1–3). If untreated, severe forms of gout can develop which not only affect multiple joints and cause joint destruction, but also display urate deposition and tophi formation in soft tissues. Urate deposits in the kidney can lead to serious complications including the formation of kidney stones and kidney failure and people with gout were found to be much more likely to die from kidney disease compared to people without gout (4). Gout is a prototypical inflammatory disease, that is driven by the activation of the innate immune system and particularly by mononuclear phagocytes (5). Resident joint macrophages remove MSU crystals by phagocytosis, which induces a local inflammatory response (6).

MSU crystal internalization activates the nucleotide binding and oligomerization domain (NOD)- leucine rich repeat (LRR)- and pyrin domain (PYD)-containing 3 (NLRP3) inflammasome resulting in the release of the highly pro-inflammatory cytokine Interleukin (IL)-1β and recruitment of immune cells (7). Hence, inhibition of this pathway is a clinically relevant treatment strategy for gout (8). The mechanism for NLRP3 inflammasome activation involves the MSU-mediated lysosomal destabilization and rupture, which causes the release of lysosomal cathepsins (9). In addition, MSU internalization also induces NLRP3 inflammasome activation through sensing of reduced intracellular K^+^ concentrations due to increased intracellular Na^+^, water influx and cellular swelling (10), the production of reactive oxygen species (ROS) (11) as well as release of ATP and the activation of purinergic signaling (12). Activation of the NLRP3 inflammasome requires two steps: priming and activation. The priming step includes the NF-κB mediated transcription of NLRP3 and IL-1β and post-translational modifications, including phosphorylation, de-phosphorylation, ubiquitination, de-ubiquitination, sumoylation and nitrosylation of inflammasome components to bring them to a “ready” state (13). The activation step requires sensing of foreign or endogenous danger signals by a pattern recognition receptor and its activation, followed by the recruitment and oligomerization of the adaptor protein ASC (apoptotic speck-like protein containing a caspase recruitment domain, CARD), and the recruitment and activation of the IL-1β cleaving cysteine protease caspase-1 (13). In addition, caspase-1 is also able to cleave gasdermin D into an N- and C-terminal fragment and the N-terminal fragment assembles into a membrane pore, which triggers an inflammatory cell death called pyroptosis (14). The formation of this inflammasome complex involves homotypic protein domain interactions between NLRP3 and ASC through their PYDs and between ASC and caspase-1 through their CARDs (15). Hence, the presence and accessibility of PYDs and CARDs is crucial for inflammasome assembly and activation.

We and others recently discovered a protein family of inflammasome inhibitors that are comprised of a single PYD, termed PYD only proteins (POPs) and were later annotated as PYD containing (PYDCs) (16). The POP family consists of four members with strong homologies to other PYD containing proteins. The first 88 amino acids of human ASC encode a PYD, which is 64% identical to POP1 (PYDC1) with conserved spacing of charged amino acids (17, 18). POP2 (PYDC2) is highly homologous to NLRP2 and NLRP7, but only 16% identical to the ASC PYD (19–22), and POP3 (PYDC5) is highly homologous to the PYD of AIM2 (23). POP4 encodes a truncated PYD from the NLRP2P pseudogene (24). Interestingly, none of these proteins are encoded in mice and evolved only in higher primates (25–27). However, human POPs are functional in mice, as determined by transgenic expression (17, 19, 20, 23). Here we show that POP1 inhibits uric acid crystal-induced NLRP3 inflammasome activation, inflammation and ameliorates gout in a mouse model.

## Results

### POP1 transgenic mice are protected from gout

**Figure 1:**
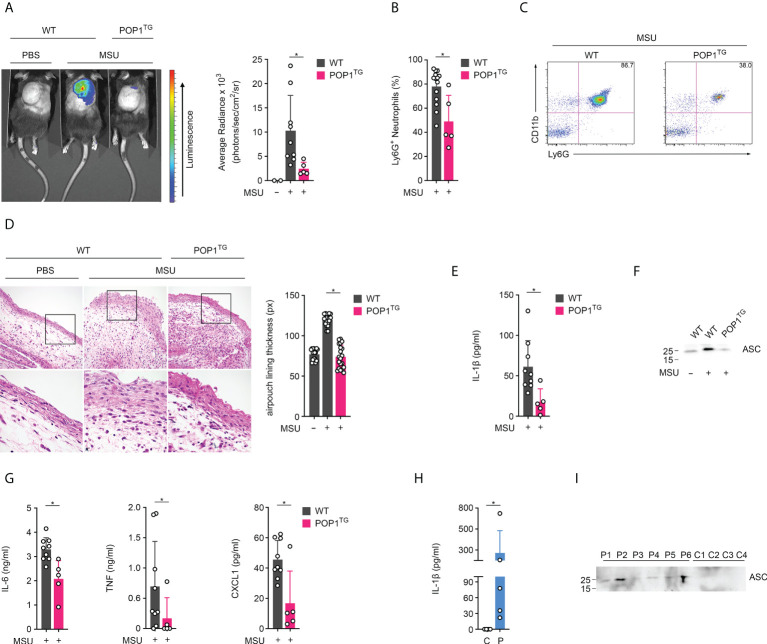
POP1 ameliorates MSU-induced inflammation in mice. **(A)** *In vivo* imaging of MPO activity correlating to MSU-induced neutrophil infiltration into subcutaneous air pouches 6h after MSU crystal injection in wild type (WT), and POP1 transgenic (POP1^TG^) mice (left) and average radiance (right) presented as photons/sec/cm^2^/sr (n = 3 - 9 ± s.d.). **(B)** Subcutaneous air pouch exudates were analyzed for CD11b^+^Ly6G^+^ neutrophils by flow cytometry in mice injected with MSU crystals (n = 5 - 14 ± s.d.). **(C)** Representative example of a dot plot gating of CD11b and Ly6G stained cells from MSU crystal injected WT and POP1^TG^ mice. **(D)** Representative H&E staining of subcutaneous air pouch lining of PBS or MSU crystal injected WT and POP1^TG^ mice 6h after PBS or MSU crystal injection (left) showing the boxed area (top) enlarged (bottom), and quantification of subcutaneous air pouch lining presented as pixel determined at five different positions (right) (n = 4 ± s.d.). **(E–G)** Subcutaneous air pouch lavage exudates were analyzed for **(E)** IL-1β, **(G)** IL-6, TNF and CXCL1 by ELISA and for **(F)** ASC by immunoblot showing a representative example. (n = 5 - 9 ± s.d.). **(H, I)** Cleared synovial fluid obtained by ankle arthrocentesis from patients diagnosed with acute gout flares **(P)** using human plasma as a control **(C)** was analyzed for **(H)** IL-1β by ELISA and **(I)** ASC by immunoblot. (n = 6 ± s.d.). *p < 0.05.

We previously reported the generation and characterization of transgenic (TG) mice expressing human POP1 from the hCD68 promoter in combination with the macrophage-specific IVS-1 enhancer, to better mimic some aspects of inflammasome regulation that evolved in humans (17). This study also demonstrated that inflammasome regulation by POP1 is comparable in humans and mice, likely aided by sufficiently conserved inflammasome components in response to infectious triggers and mutation-driven inflammasome responses. However, the response to sterile inflammasome triggers, such as crystalline DAMPs, has not yet been investigated. Deposition of MSU crystals into articular joints and bursal tissue and phagocytosis by resident macrophages results in neutrophil influx and consequently the acute joint inflammation characteristic of gout. The murine subcutaneous air pouch resembles this bursa-like space with a synovial-like membrane characteristic of the synovial lining (28). Injection of MSU crystals into this air pouch therefore recapitulates the MSU response present in the joints of gout patients and activates the NLRP3 inflammasome (7, 19, 29). Injection of MSU crystals, but not PBS into the synovium-like subcutaneous air pouch of WT mice therefore resulted in acute neutrophil infiltration after 6 hours, as quantified by *in vivo* imaging using a luminescent probe selective for neutrophil myeloperoxidase (MPO) activity (30). In contrast, MPO activity was almost completely absent in MSU crystal-injected POP1^TG^ mice, indicating reduced inflammation (**Figure 1A**). Analysis of the synovium-like subcutaneous air pouch exudate by flow cytometry 6 hours after MSU crystal injection also demonstrated a reduced number of CD11b^+^Ly6G^+^ neutrophils in POP1^TG^ mice (**Figure 1B**), showing a representative result (**Figure 1C**). H&E staining revealed that the thickness of the synovial-like lining of the air pouch, which displayed leukocytic infiltrates and marked swelling 6 hours after MSU crystal injection in WT mice, was markedly reduced in MSU crystal injected POP1^TG^ mice (**Figure 1D**). Neutrophil infiltration in response to sterile triggers is driven by inflammasome activation and IL-1β (31). Indeed, further analysis of the synovium-like subcutaneous air pouch exudate showed reduced IL-1β levels in POP1^TG^ mice compared to WT mice (**Figure 1E**). NLRP3 inflammasome activation does not only result in cytokine release, but also pyroptosis and the release of danger signals, including polymerized ASC particles, which propagate and perpetuate inflammasome responses to bystander cells upon phagocytosis (32, 33). The synovium-like subcutaneous air pouch exudate from MSU crystal injected POP1^TG^ mice also revealed reduced levels of released ASC, as determined by immunoblot (**Figure 1F**). Although, MSU crystal-induced inflammasome activation does not directly affect release of other cytokines, exudates from MSU-injected POP1^TG^ mice also displayed reduced levels of the pro-inflammatory IL-6, TNF and CXCL1 (**Figure 1G**). A similar observation was previously made using an NLRP3 inhibitor in a gout model, which also suppressed IL-6 and CXCL1 in synovial tissue after MSU crystal injection (34). To demonstrate that the inflammasome activation that we observed in the murine air pouch model also occurs in gout patients, we determined the presence of IL-1β and ASC in the cleared synovial fluids obtained by ankle arthrocentesis from patients diagnosed with acute gout flares and the presence of crystals. Plasma from healthy subjects was used as a specificity control for the assay, which confirmed the presence of IL-1β (**Figure 1H**) and ASC (**Figure 1I**) in gout synovial fluids. Overall, these results demonstrate that POP1 expression in macrophages regulates NLRP3 inflammasome activation and results in an ameliorated inflammatory response to MSU crystals *in vivo*.

### POP1 expression reduces MSU crystal induced joint inflammation

**Figure 2:**
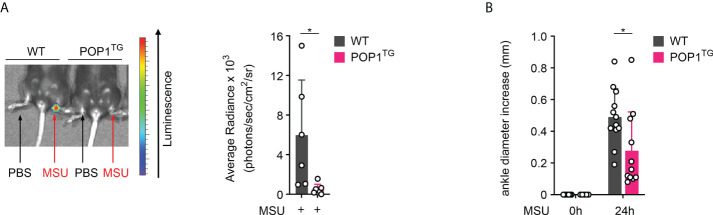
POP1 ameliorates MSU-induced joint inflammation in mice. **(A)** *In vivo* imaging of MPO activity correlating to PBS (left ankle) or MSU (right ankle)-induced neutrophil infiltration into the hind limb ankle joint in wild type (WT) and POP1 transgenic (POP1^TG^) mice 24h after PBS or MSU crystal injection (left) and average radiance (right) presented as photons/sec/cm^2^/sr (n = 5 - 6 ± s.d.). **(B)** The ankle diameter was measured with a caliper before and 24h after MSU crystal injection into the hind limb ankle joint in WT and POP1^TG^ mice (n = 11 - 12 ± s.d.). *p < 0.05.

MSU crystal deposition usually affects a single joint progressing to MSU crystal tophi, progressive arthritis of multipole joints and joint destruction (35). To directly assess the consequence of POP1 expression in macrophages on joint inflammation, we injected MSU crystals or PBS into the right and left hind limb ankle joint, respectively in WT and POP1^TG^ mice. While MSU crystals caused a strong inflammatory response, as indicated by *in vivo* imaging of MPO activity, this response was also almost completely prevented in POP1^TG^ mice 24 hours post MSU crystal injection (**Figure 2A**). MSU crystal-induced inflammation also resulted in edema and increased ankle circumference 24 hours after MSU crystal injection in WT mice, but also this response was significantly reduced in POP1^TG^ mice (**Figure 2B**), indicating that the ameliorated inflammatory response to sterile NLRP3 activators is independent of the target organ and that POP1 has an important role in preventing excessive inflammatory responses and the pathology of gout.

### POP1 expression reduces MSU crystal induced cytokine release in BMDM

**Figure 3:**
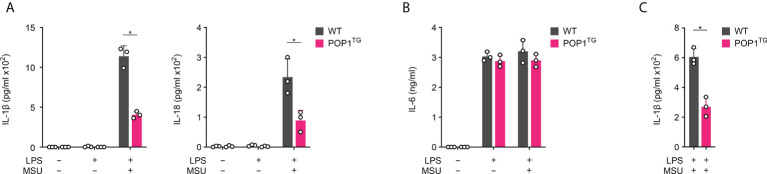
POP1 inhibits inflammasome-dependent cytokine release in BMDM. **(A, B)** Wild type (WT) and POP1 transgenic (POP1^TG^) BMDM were primed with LPS and treated with MSU crystals as indicated and culture supernatants (SN) were analyzed for secreted **(A)** IL-1β and IL-18 and **(B)** IL-6 by ELISA (n = 3 ± s.d.). **(C)** Peritoneal macrophages were treated as above, and SN were analyzed for secreted IL-1β by ELISA (n = 3 ± s.d.). *p < 0.05.

We previously demonstrated that human POP1 has a conserved role in mouse macrophages in response to infectious triggers, such as LPS (17), but its role in response to sterile, endogenous danger signals is largely unknown. Due to the ameliorated *in vivo* response to MSU crystals, we investigated inflammasome mediated cytokine release in BMDM. While activation of LPS primed macrophages with MSU crystals resulted in release of IL1-β in BMDM isolated from WT mice, this response was markedly reduced in BMDM isolated from POP1^TG^ mice (**Figure 3A**). LPS priming alone did not cause any release of IL-1β. Inflammasome activation does not only cause release of IL-1β, but also release of the related IL-18. Accordingly, POP1^TG^ BMDM also showed significantly reduced release of IL-18 in response to MSU crystals in LPS primed macrophages (**Figure 3A**). Other pro-inflammatory cytokines, such as IL-6 or TNF, are regulated by NF-κB, but do not require the inflammasome for release. IL-6 release therefore required macrophage priming with LPS, but activation with MSU crystals did not further elevate the release of IL-6. In agreement with an inflammasome regulatory function of POP1, POP1^TG^ BMDM released IL-6 comparably to WT BMDM (**Figure 3B**). This is contrary to the reduced IL-6 and TNF levels observed in response to MSU crystal injection into the synovium-like subcutaneous air pouch of POP1^TG^ mice, indicating that the reduced cytokine levels observed *in vivo* during gout are likely secondary to the POP1-mediated reduction of pro-inflammatory cell infiltration, rather than a direct effect of POP1 on NF-κB mediated cytokine expression. Further, POP1 reduced the MSU crystal induced release of IL-1β not only in BMDM, but also in LPS primed and MSU crystal activated peritoneal macrophages (**Figure 3C**).

### POP1 inhibits MSU crystal induced inflammasome assembly and caspase-1 activation

**Figure 4:**
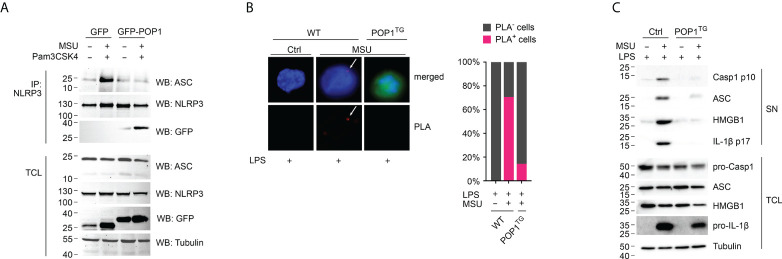
POP1 inhibits MSU crystal-induced NLRP3 inflammasome assembly and caspase-1 activation. **(A)** Immunoprecipitation (IP) with immobilized anti-NLRP3 antibodies from either untreated or primed (Pam3CSK4) and activated (MSU crystals) GFP control and GFP-POP1 THP-1 cell lysates as indicated. Immunoblot of IPs and total cell lysates (TCL) for ASC, NLRP3, GFP and as a loading control tubulin. **(B)** Proximity ligation assay (PLA) between NLRP3 and caspase-1 in LPS primed and MSU crystal activated WT and POP1 transgenic (POP1^TG^) BMDM. DNA (blue), POP1 (green) and an arrow marks the PLA signal (red), showing a representative result (left) and quantification of PLA^+^ cells of three randomly selected fields of views (right). **(C)** LPS primed WT and POP1^TG^ BMDM were activated with MSU crystals and culture supernatants (SN), and total cell lysates (TCL) analyzed by immunoblot for active caspase-1 p10, pro-caspase-1, ASC, HMGB1, mature IL-1β p17, pro-IL-1β, and tubulin as indicated, showing a representative result.

NLRP3 inflammasome activation results in inflammasome assembly and caspase-1 activation, which is responsible for maturation and release of IL-1β and IL-18 (36, 37). The process of caspase-1 activation requires the ASC mediated recruitment of pro-caspase-1 to activated NLRP3, polymerization of pro-caspase-1 and caspase-1 proteolysis through a proximity-induced auto-activation mechanism (38–41). PYD-mediated recruitment of ASC to activated NLRP3 is a key step in inflammasome assembly, resulting in ASC polymerization that subsequently results in caspase-1 activation (39, 40). We therefore determined this essential recruitment of ASC to NLRP3 by immunoprecipitating NLRP3 from GFP control and GFP-POP1 expressing THP-1 cells, which we characterized earlier, and which are commonly employed for inflammasome studies (17). While ASC co-precipitated with NLRP3 only in primed and MSU activated control cells and not in untreated cells, ASC did not co-purify with NLRP3 in primed and MSU-activated POP1 expressing cells (**Figure 4A**). We earlier demonstrated that POP1 binds to the PYD of ASC to prevent its recruitment to NLRP3 (17), and our results further demonstrate that POP1 also bound to NLRP3 in primed and MSU-activated cells, but not in untreated cells and that this interaction coincided with loss of ASC binding to NLRP3 (**Figure 4A**). The POP1 interaction with NLRP3 upon MSU activation likely contributed to prevent the ASC recruitment to NLRP3. Since ASC is necessary for bridging the recruitment of caspase-1 to NLRP3, we also determined NLRP3 inflammasome assembly in LPS primed and MSU crystal activated intact BMDM by the Proximity Ligation Assay (PLA) between NLRP3 and caspase-1. PLA^+^ signals therefore represent the assembled NLRP3 inflammasome. While MSU treatment of WT BMDM resulted in the characteristic punctate PLA signal in ~70% of cells as determined by microscopy, only ~15% of POP1^TG^ BMDM showed a PLA signal (**Figure 4B**), demonstrating that indeed the NLRP3 inflammasome assembly is reduced, but not completely prevented by POP1. NLRP3 inflammasome assembly is required for caspase-1 activation and active caspase-1 as well as the polymerized ASC adaptor are released by unconventional protein secretion during macrophage pyroptosis (32, 33, 42). Activation of LPS primed WT BMDM with MSU crystals therefore caused proteolysis of caspase-1 and release of the active p10 fragment into the culture supernatant (SN). However, this process was almost completely prevented in POP1^TG^ BMDM, resulting in lack of active caspase-1 p10 in the culture SN of LPS-primed and MSU crystal activated POP1^TG^ BMDM (**Figure 4C**). However, expression of full-length caspase-1 in total cell lysates (TCL) was not altered by POP1. Similarly, ASC is present in LPS primed and MSU crystal activated SN of WT BMDM, where it is involved in propagation and perpetuation of inflammasome responses (39, 40, 43), but is absent in the SN of POP1^TG^ BMDM (**Figure 4C**). POP1 expression also did not affect ASC expression in TCL. Similarly, the danger signal HMGB1, which is released by pyroptosis (44), is only present in the SN of WT but not POP1^TG^ BMDM, matching the release of mature IL-1β p17, also without altering expression in TCL (**Figure 4C**). Therefore, POP1 prevents MSU crystal induced NLRP3 inflammasome assembly, caspase-1 activation and the release of danger signals and cytokines that require inflammasome activation.

### POP1 expression inhibits MSU crystal induced cytokine release in THP-1 cells

**Figure 5:**
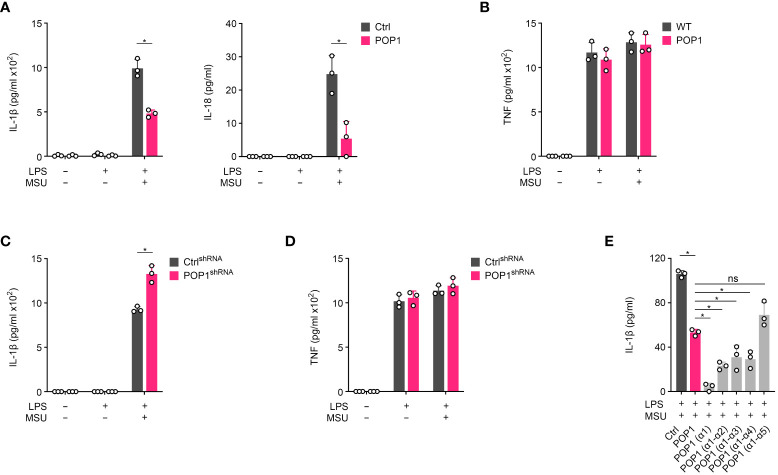
POP1 inhibits inflammasome-dependent cytokine release in human macrophages. **(A, B)** Control (Ctrl) and POP1 expressing THP-1 cells (POP1) were primed with LPS and treated with MSU crystals as indicated and culture supernatants (SN) were analyzed for secreted **(A)** IL-1β and IL-18 and **(B)** TNF by ELISA (n = 3 ± s.d.). **(C, D)** Ctrl shRNA and POP1 shRNA expressing THP-1 cells were primed and treated with MSU crystals as above and culture SN were analyzed for secreted **(C)** IL-1β and **(D)** TNF by ELISA (n = 3 ± s.d.). **(E)** LPS primed Control (Ctrl) THP-1 cells or THP-1 cells stably expressing POP1 or the indicated α helices of POP1 were activated with MSU crystals and culture supernatants were analyzed for secreted IL-1β by ELISA (n = 3 ± s.d.). *p < 0.05, ns, non specific.

To ensure that POP1 functions comparably in human and mouse macrophages, we utilized the POP1 expressing THP-1 cells. MSU crystal activation of LPS primed THP-1 cells also resulted in the release of IL-1β, which was significantly reduced in THP-1 cells expressing POP1 (**Figure 5A**). We observed a similar result for the MSU crystal activated release of IL-18 in LPS primed THP-1 cells (**Figure 5A**). However, the inflammasome independent release of TNF in response to LPS priming or LPS priming followed by MSU crystal activation was not altered in POP1 expressing THP-1 cells, comparable to what we observed for IL-6 in BMDM (**Figure 5B**), further supporting the inflammasome regulatory function of POP1. Furthermore, LPS primed THP-1 cells with silenced POP1, which we characterized earlier (17), correspondingly showed elevated release of IL-1β in response to MSU crystal activation, when compared to control cells (**Figure 5C**), while release of TNF was again not affected (**Figure 5D**). The PYD is characterized by six α-helical domains that assemble the binding interface, and POP1 is structurally highly similar to other PYDs (45, 46). To potentially identify POP1 domains sufficient to inhibit the NLRP3 inflammasome response, reminiscent to what has been earlier identified for the related POP2, where α1 is sufficient for NF-κB and inflammasome inhibition (47), we generated THP-1 cells expressing POP1 mutants consisting of truncated individual α-helices, based on the POP1 crystal structure (46). Earlier, mutagenesis identified that α helix 2 and α helix 3 of POP1 play an important role in the interaction with ASC (48), but reminiscent to POP2, the α helix 1 of POP1 was also sufficient to block IL-1β release in response to MSU crystal activation of LPS primed THP-1 cells (**Figure 5E**). The α helix 1 was even more efficient than full length POP1 in inhibiting IL-1β release, while α helices 1-2, 1-3 and 1-4 showed an intermediate effect, and α helices 1-5 were comparable to full length POP1 comprised of α helices 1-6 (**Figure 5E**). We identified earlier that POP1 is phosphorylated (18), which may affect the inhibitory function of full length POP1. These results confirm that the inflammasome inhibitory function of POP1 is comparable in human and mouse macrophages, likely facilitated by the highly conserved sequences of core inflammasome proteins.

### Cell penetrating POP1 peptides ameliorate MSU crystal induced inflammation

**Figure 6:**
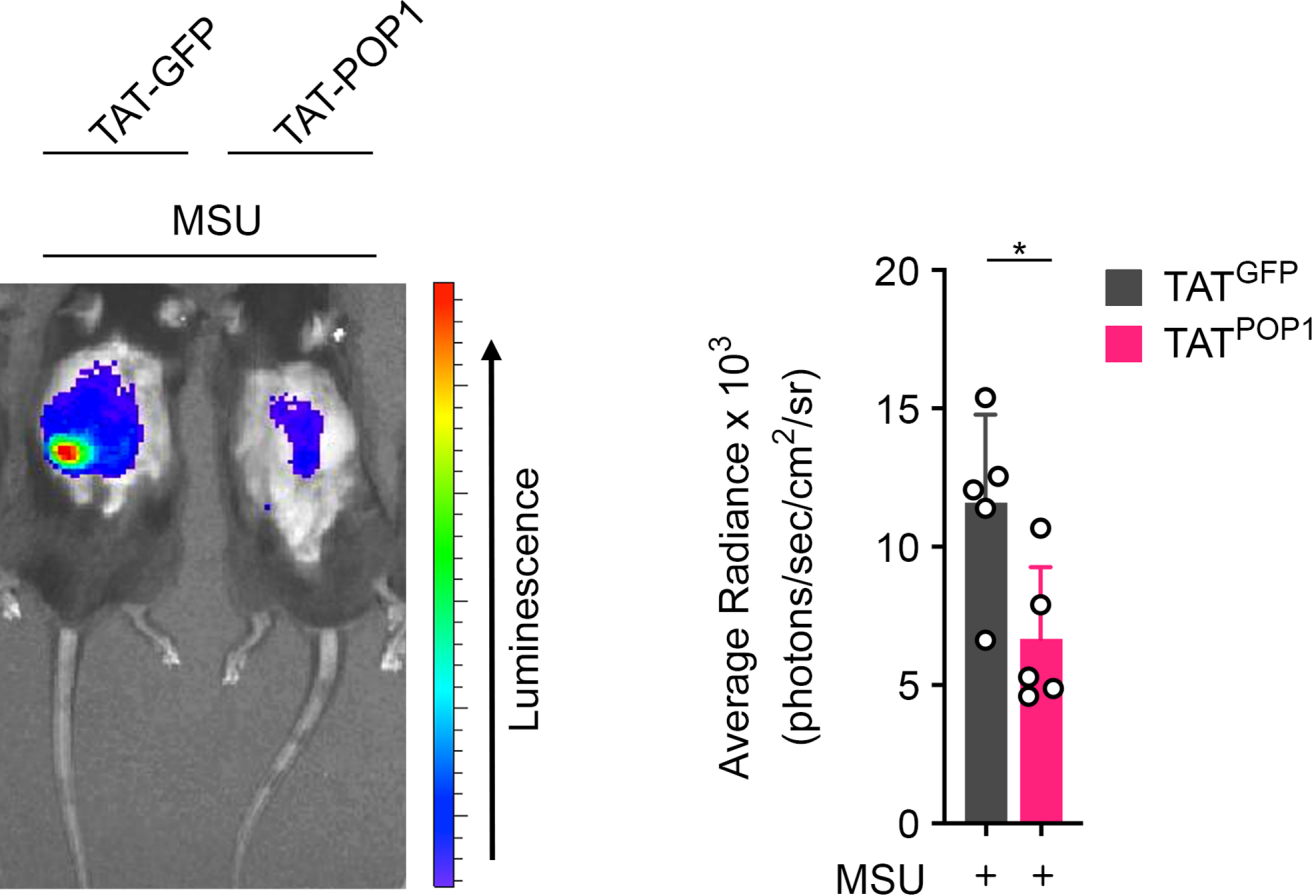
Cell penetrating POP1 ameliorates gout. *In vivo* imaging of MPO activity correlating to MSU-induced neutrophil infiltration into subcutaneous air pouches 4h after MSU crystal injection in wild type mice injected with either TAT-GFP or TAT-POP1 30 minutes prior MSU crystal injection (left) and average radiance (right) presented as photons/sec/cm^2^/sr (n = 5 ± s.d.). *p < 0.05.

Cell-penetrating peptides are frequently employed for the delivery of molecules targeting intracellular signaling pathways (49) and we previously demonstrated that cell penetrating POP1 is able to ameliorate sepsis (17). Since, POP1^TG^ mice were protected from gout, we utilized cell penetrating POP1 to investigate its efficacy for the treatment of gout in a proof-of-principle study. Recombinant, endotoxin free POP1 or GFP control fused to the cell-penetrating HIV TAT sequence (TAT-POP1 and TAT-GFP) were i.p. injected, followed by MSU crystal injection into the subcutaneous air pouch. Mice were then analyzed for infiltrating neutrophils by *in vivo* MPO imaging, as above. While TAT-GFP injected mice showed a robust MPO signal, mice injected with TAT-POP1 displayed a significantly attenuated MPO signal (**Figure 6**), indicating reduced numbers of infiltrating neutrophils and ameliorated inflammation, reminiscent to transgenic POP1 expression.

## Discussion

NLRP3 inflammasome assembly is based on the core concept of NLRP3 oligomerization initiating sequential nucleated polymerization steps. Activation and oligomerization of NLRP3 nucleate prion-like polymerization of ASC, which in turn nucleates the polymerization of caspase-1 filaments that facilitate the close proximity necessary for caspase-1 activation (39, 40, 43). We previously demonstrated that this first step of nucleated ASC polymerization is regulated by members of the POP family by competitive binding to the PYD of either ASC or upstream sensors, thereby ameliorating inflammasome responses (18, 21–23). As all four POP family members only evolved in higher primates, they are absent from most species, including mice, which suggests an additional level of regulation for the prevention of excessive inflammation in higher organisms (25–27). However, there are functional differences between the individual POP members. POP1, which is most similar to the PYD of ASC, inhibits inflammasome assembly in macrophages by binding to the PYD of ASC, thereby preventing NLRP3-ASC interactions and subsequently ASC polymerization (17, 18). POP2 also binds to ASC and inhibits inflammasome assembly, but it also inhibits NF-κB signaling and pro-inflammatory cytokine productions and enhances IFN-γ to establish anti-bacterial immunity (19–22). POP3 specifically regulates DNA induced inflammasomes by binding to AIM2 and IFI16 and also regulates type I interferon responses (23). In addition, a related truncated protein, POP4 originating from NLRP2P encodes only the first two α helices, inhibits NF-κB and also evolved in higher primates (24). In spite of their absence from the mouse genome, transgenic expression of human POP family members in mice from a macrophage specific- or an endogenous promoter recapitulates their inflammasome inhibitory function *in vivo* where they ameliorate inflammation and improve protection against bacterial infection (17, 19, 20, 23).

We previously showed that POP2 interferes with MSU crystal induced inflammasome responses and ameliorates gout (19), but the role of POP1 in the pathology of gout has not been investigated yet. Therefore, we analyzed whether POP1 is also able to regulate this response.

The ubiquitous metabolite uric acid is released from dying and damaged cells and in its crystalline form of monosodium urate, acts as a potent pro-inflammatory danger signal (50). Deposition of these MSU crystals in joints and tissues causes usually self-limiting inflammation and gout (51). The main mechanism by which MSU crystals cause inflammation is through activating the NLRP3 inflammasome and therefore, blocking NLRP3 or IL-1β is an effective treatment for severe or refractory gout (7, 8, 34, 52). The prevalence of gout is steadily increasing due to risk factors that include diet, obesity and an aging population and is more common in men and currently 5.9% of the US male population suffer from gout (53). While gout flares are usually self-resolving, it eventually may lead to permanent joint damage and further complications, including impaired vision, chronic kidney disease and heart disease (51, 54). However, high levels of uric acid and deposition of MSU crystals into joints and tissues does not always cause gout, suggesting the existence of regulators that shape MSU crystal induced inflammation. We identified that reminiscent to POP2, also POP1 inhibits uric acid crystal-induced NLRP3 inflammasome activation and ameliorates gout. Hence, POP1 is a potent regulator of inflammatory responses to MSU crystals. Mechanistically, we demonstrate that POP1 prevents MSU crystal induced NLRP3 inflammasome assembly, the resulting activation of caspase-1 and the release of inflammasome dependent cytokines and danger signals. We earlier identified that POP1 binds to the PYD of ASC to prevent recruitment of ASC to activated NLRP3, thereby preventing inflammasome assembly (17). We now provide evidence that POP1 also binds to NLRP3 in primed and MSU-activated cells, but not in untreated cells, which contributes to prevent recruitment of ASC. As POP1 expression is comparable in untreated and primed and MSU activated cells but POP1 only binds to NLRP3 after priming and activation, this interaction may be regulated by posttranslational modifications, reminiscent to other NLRP3 binding proteins (13). Strikingly, expression of POP1 exclusively in macrophages is sufficient for reducing inflammation. While POP1 specifically prevents inflammasome-mediated cytokine release in isolated mouse macrophages, a broader inhibition of pro-inflammatory cytokines and chemokines was observed in the *in vivo* gout model. We find reduced IL-1β and less infiltrating immune cells in POP1^TG^ mice which likely contributes to the reduced cytokine and chemokine levels. A similar observation was made upon NLRP3 inhibition in an *in vivo* gout model (34). However, *in vivo* responses to MSU crystals may also show differences in cytokine and chemokine levels depending on the time point of analysis.

Our study provides further support that POP1 is an important regulator of sterile inflammatory responses by targeting the MSU crystal induced NLRP3 inflammasome. Since POP1 expression in mice that otherwise lack POP1 ameliorated gout, it is conceivable that dysregulated expression of POP1 may affect the susceptibility for developing gout flares. Employing our previously characterized cell-penetrating TAT-POP1, we demonstrated that even ectopic delivery of POP1 reduced MSU crystal-mediated inflammation *in vivo*, and truncated cell penetrating POP1 peptidomimetics may potentially be even more effective than POP1 in inhibiting the NLRP3 inflammasome. Possible, the earlier identified phosphorylation of POP1 may regulate its function, such as folding and its interaction with ASC, which may not affect a shorter peptide. Although, POP1 has several patches that interact with ASC (46, 48), occupying any of individual patch may be sufficient to impair ASC interactions necessary for inflammasome assembly. Whether truncated cell penetrating POP1 peptides are also more efficient in ameliorating MSU crystal induced NLRP3 inflammasome responses *in vivo* will require future studies. Our results further emphasized the importance of tightly controlling essential PYD-PYD interactions between ASC and NLRP3 in order to prevent unwanted or excessive NLRP3 inflammasome responses. Therefore, POP1 is a regulatory protein that evolved in humans in order to prevent excessive and uncontrolled inflammatory responses to endogenous danger signals, providing novel insights into the unique aspects of the human innate immune system.

## Materials and methods

### Animals

TgN(*CD68-POP1*) (POP1^TG^) mice were generated on the C57BL/6J strain as previously described (17) and were housed in a specific pathogen-free animal facility. All experiments were performed on age and gender-matched 8–12 week old mice conducted according to procedures approved by the Northwestern University Committee on Use and Care of Animals.

### Air pouch gout model

8- to 12-week-old WT and POP1^TG^ mice had their back shaved under anesthesia and 3 ml of sterile air was injected subcutaneously on the back to generate an air pouch using a 25-gauge syringe. Three days later, subcutaneous air pouches were re-inflated with 2 ml of sterile air. Mice were randomly selected for subcutaneous air pouch injection with PBS or MSU crystals (3 mg) on day 6. After 6h, mice were injected i.p. with a XenoLight Rediject Inflammation probe (200 mg/kg, PerkinElmer) and *in vivo* bioluminescence was captured by imaging (IVIS Spectrum, PerkinElmer) 10 min after injection with a 5 min exposure time on anesthetized mice. Images were quantified with Living Image software (PerkinElmer, RRID : SCR_014247). Subcutaneous air pouch lavage exudates were collected 6h after MSU crystal injection. Cells were recovered from the lavage exudate and were analyzed by flow cytometry and cytokine levels were quantified by ELISA.

### MSU crystal ankle injection

8- to 12-week-old WT and POP1^TG^ mice were injected with 20 μl PBS or MSU crystals (100 μg/ml) using a Hamilton syringe into the left and right hind limb ankle joint and 24h later, injected i.p. with XenoLight Rediject Inflammation probe (200 mg/kg, PerkinElmer) and imaged as above. Ankle circumference was measured with a caliper before injection and 24 h after injection.

### Cells

Mouse bone marrow cells were flushed from femurs, differentiated into BMDMs with L929 cell-conditioned medium (25%; ATCC, #CCL-1, RRID : CVCL_0462) in DMEM medium, supplemented with 10% heat inactivated fetal bovine serum (Gibco), 5% horse serum (Gibco) and analyzed after 7 days. Peritoneal macrophages (PM) were obtained by peritoneal lavage. THP-1 cells were obtained from the American Type Culture Collection (ATCC, #TIB-202, RRID : CVCL_0006) and maintained in RPMI media supplemented with 10% Fetal bovine serum (Gibco), 1 mM sodium pyruvate, 0.05 mM 2-mercaptoethanol, 1 mM HEPES buffer and 1% penicillin and streptomycin. Lenti-X human embryonic kidney (HEK) 293 cells (632180, Takara Bio) were maintained in DMEM high glucose media containing 10% FBS, 100 IU/ml penicillin and 1 mg/ml streptomycin. Cells were routinely tested for mycoplasma contamination (MycoAlert, Lonza). All THP-1 cell experiments were performed in the absence of PMA differentiation. Cells were seeded in 1% FBS and antibiotic free media at a density of 2×10^5^ cells in a 24-well plate and were primed with ultra pure *E. coli* LPS (0111:B4, *In vivo*gen, 100 ng/ml) for 16h and treated with MSU (90 μg/ml) for 6h. Cell culture supernatants were harvested for enzyme-linked immunosorbent assays (ELISAs) or immunoblot after stimulation.

### Generation of stable POP1 domain expressing cells

EGFP-POP1 expressing and POP1 shRNA expressing THP-1 cells were described earlier (17). POP1 truncated mutants were generated by cloning gBlocks (Integrated DNA Technologies) into modified pLex lentivirus vectors in frame with an N-terminal enhanced green fluorescent protein (EGFP): h1 (aa 1-16), h1-h2 (aa 1-33), h1-h3 (aa 1-49), h1-h4 (aa 1-61), h1-h5 (aa 1-79). Recombinant lentivirus was produced in Lenti-X HEK293 cells by Xfect-based transfection (Takara Bio) with the viral packaging plasmids psPAX2 (a gift from Didier Trono, Addgene plasmid # 12259, http://n2t.net/addgene:12259) and psPAX2 (a gift from Didier Trono, Addgene plasmid # 12260, http://n2t.net/addgene:12260), followed by 0.45 μm filtration of virus-containing culture SN. Wild type THP-1 cells that were also used for expression of EGFP-POP1, were transduced with lentiviral particles encoding the truncated POP1 mutants in the presence of polybrene (0.45 μg/ml) and MISSION ExpressMag magnetic beads (Millipore-Sigma). Cells were Puromycin-selected (1 μg/ml, Santa Cruz Biotechnology) 48h post infection for 2 weeks, sorted by flow cytometry and expression was verified by immunoblot.

### Immunoprecipitation and immunoblotting

*Immunoprecipitation:* THP-1 cells were untreated or primed with Pam3CSK4 (*In vivo*gen, 1 μg/ml, tlrl-pms) for 3h, activated with MSU crystals (90 μg/ml, *In vivo*gen, tlrl-msu) for 0.5h, washed and lysed in 50 mM HEPES, pH 7.4, 50 mM NaCl, 10% glycerol, 2 mM EDTA, 0.5% Triton X-100, supplemented with protease inhibitors. Cleared lysates were subjected to IP by incubating with immobilized rabbit monoclonal anti-NLRP3 antibodies (Cell Signaling, D4D8T, RRID : AB_2722591) on agarose A/G beads (Santa Cruz Biotechnology) for 4–16h at 4°C. Following extensive washing with lysis buffer, bound proteins in Laemmli sample buffer were separated by SDS/PAGE and analyzed by immunoblot.

*Immunoblot:* Total cell lysates or immunoprecipitated proteins were separated on polyacrylamide gels and transferred onto polyvinylidene fluoride membranes (Millipore). Membranes were blocked (5% non-fat dry milk, 0.1M Tris-buffered saline, pH 7.4, 0.1% Tween 20) for 1h at room temperature followed by incubation with primary antibodies for 12-16h at 4°C as indicated: rabbit polyclonal antibody to ASC (AdipoGen, AL177, RRID : AB_2885200; 1:1000) or for IPs with HRP-conjugated mouse monoclonal antibody to ASC (Santa Cruz Biotechnology, B-3, sc-514414, RRID : AB_2737351, 1:2000), HRP-conjugated rabbit monoclonal antibody to GFP (Cell Signaling, D5.1, RRID : AB_1281301, 1:1000), mouse monoclonal antibody to NLRP3 (Adipogen, AG-20B-0014-C100, RRID : AB_2885199, 1:1000), mouse monoclonal antibody to caspase-1 (Santa Cruz Biotechnology, 14F468, RRID : AB_781816; 1:200), rabbit polyclonal antibody to IL-1β (Santa Cruz Biotechnology, SC7884, RRID : AB_2124476; 1:50), and Tubulin (DSHB, AA12.1, RRID : AB_579794; 1:5). Membranes were washed and incubated with HRP-conjugated secondary antibodies, including goat anti-Rabbit IgG (H+L) HRP (Invitrogen, 31460; 1:10000) and goat anti-Mouse IgG (H+L) HRP (Invitrogen, 32430; 1:10000) for 1h at room temperature and proteins were visualized by ECL detection (Super Signal West Femto, ThermoFisher Scientific) and digital image acquisition (Ultralum Omega 14vR). Before re-probing membranes, bound antibodies were stripped (0.1 M Glycine, 2% SDS) and membranes were washed (0.1 M Tris-buffered saline, pH 7.4, 0.1% Tween 20).

### Uric acid crystal preparation

Endotoxin free MSU crystals were prepared by crystallization of a supersaturated uric acid solution as described earlier (19). Briefly, 1.68 gm of uric acid (Sigma) was added in 25 mM sodium hydroxide solution and left for mixing on a rotator for 18-20h at room temperature. MSU crystals were harvested by filtration through a 0.22-μM filter, washed times with sterile ice cold PBS, followed by one ethanol (70% v/v) wash. MSU crystals were air dried, UV sterilized, resuspended in PBS (100 mg/ml), verified for needle shape by microscopy and tested on ASC knock down cells (55) for specificity.

### Flow cytometry

Cells obtained from air pouch lavage fluid were stained with anti-Ly6G (BD Biosciences, 1A8) and anti-CD11b (eBioscience, M1/70). Data were acquired on a BD LSR II flow cytometer (BD Biosciences). Compensation and analysis of the flow cytometry data were performed using FlowJo software (TreeStar).

### Immunohistochemistry

Synovium-like subcutaneous air pouch lining tissues samples were harvested 6h after injection of MSU crystals into the subcutaneous air pouch, fixed in formalin, embedded in paraffin blocks and stained with hematoxylin and eosin and images captured by microscopy.

### ELISA

Cell culture supernatants or synovium-like subcutaneous air pouch exudates were analyzed for IL-1β (Invitrogen), IL-18 (R&D), IL-6 (BD Biosciences), TNF (Invitrogen) or CXCL1 (R&D Systems) secretion by ELISA as per the manufacturer’s instructions.

### Proximity ligation assay

PLA (Duolink PLA, Millipore-Sigma) was performed according to the manufacturer’s instructions. Briefly, THP-1 cells were differentiated using phorbol 12-myristate-13-acetate (PMA, 20 nM, 16h) on coverslips, washed in PBS and rested for 48h. Following treatment, cells were washed with PBS, fixed with 3.7% paraformaldehyde for 10 min, permeabilized with 0.2% Triton X-100 for 10 min and washed with PBS at room temperature. All incubations were performed in a humidified chamber at 37°C. Cells were blocked with Duolink Blocking Solution for 1h, followed by incubation with primary antibodies in Duolink Antibody Diluent for 2h (rabbit polyclonal antibody to NLRP3, Cell Signaling Technology D4D8T, RRID : AB_2722591, mouse monoclonal antibody to caspase-1, Santa Cruz Biotechnology, 14F468, RRID : AB_781816), incubated with PLUS and MINUS PLA probes, washed (Buffer A) at room temperature, incubated with Ligase for 30 min, washed (Buffer A), incubated with polymerase for 100 min, washed (Buffer B) and mounted on slides using a Duolink *In Situ* Mounting Medium with DAPI. Cells were analyzed by immunofluorescence microscopy (Nikon TE200E2) and image deconvolution.

### Cell-penetrating recombinant proteins

6xHIS-POP1 and a 6xHIS-GFP cDNAs were fused with the HIV TAT sequence, purified from *E. coli*, detoxified by anion exchange as described earlier (17). Subcutaneous air pouches were generated in 12-week-old male WT mice as described above and were randomly selected for i.p. injection with TAT- GFP or TAT-POP1 (40 mg/kg) for 30 min prior MSU injection (3 mg) into the subcutaneous air pouch and were quantified for MPO activity *in vivo* as described above 3h after MSU crystal injection.

### Human gout patients

Patients were diagnosed with acute gout flares by the Northwestern Medicine Rheumatology clinic, according to the guidelines by the American College of Physicians (56). De-identified synovial fluids were collected by ankle arthrocentesis and the presence of MSU crystals in the synovial fluid was confirmed by polarizing microscopy. De-identified peripheral blood was collected by venipuncture into EDTA-containing tubes for isolation of plasma by centrifugation. 40 μl of synovial fluids or plasma were cleared by centrifugation and analyzed by ELISA and immunoblot. All studies were approved by the Northwestern University Institutional Review Board.

### Statistics

All experiments have been repeated at least three times when showing a representative result and graphs were prepared in Prism 9 (GraphPad, RRID : SCR_002798) and represent the mean ± s.d. A standard two-tailed unpaired t-test was used for statistical analysis of two groups with all data points showing a normal distribution. Values of P <0.05 were considered significant and marked by an asterisk. The investigators were not blinded to the genotype of the mice/cells.

## Data availability statement

The original contributions presented in the study are included in the article/Supplementary Material. Further inquiries can be directed to the corresponding authors.

## Ethics statement

The studies involving human participants were reviewed and approved by Northwestern University Institutional Review Board. The patients/participants provided their written informed consent to participate in this study. The animal study was reviewed and approved by Northwestern University Committee on Use and Care of Animals.

## Author contributions

LA designed and performed most of the experiments, analyzed data and prepared preliminary figures and obtained funding. SD performed the inflammasome complex purification. MI contributed to the IVIS imaging and flow cytometry. Q-QH and RP contributed to the ankle gout model and provided expertise. RR purified the cell penetrating proteins. AD and CS obtained funding, conceived and supervised the study, performed some experiments, generated figures and wrote the manuscript. All authors provided critical review of the manuscript. All authors contributed to the article and approved the submitted version.

## Funding

This work was supported by the National Institutes of Health (AI099009 and AR064349 to CS, AI134030, AI140702, AI165797 and AI120625 to CS and AD), the American Heart Association 18CDA34110296 to LA and Pfizer WI223665 to CS. The funder was not involved in the study design, collection, analysis, interpretation of data, the writing of this article or the decision to submit it for publication.

## Acknowledgments

In Memoriam of Dr. Calvin R. Brown, Jr., MD, who graciously supported our research by saving remnant synovial fluids after clinical diagnosis. psPAX2 and pMD.2G were a gift from Didier Trono.

## Conflict of interest

The authors declare that the research was conducted in the absence of any commercial or financial relationships that could be construed as a potential conflict of interest.

## Publisher’s note

All claims expressed in this article are solely those of the authors and do not necessarily represent those of their affiliated organizations, or those of the publisher, the editors and the reviewers. Any product that may be evaluated in this article, or claim that may be made by its manufacturer, is not guaranteed or endorsed by the publisher.
